# Congenital splenic cyst –case study

**Published:** 2011-02-25

**Authors:** C Iorga, V Strambu, F Popa, C Puscu, P Radu

**Affiliations:** * ‘Carol Davila’ University of Medicine and Pharmacy, BucharestRomania; ** ‘Sfantul Pantelimon’ Clinical Emergency Hospital Bucharest, General Surgery Clinic, BucharestRomania

**Keywords:** splenectomy, pseudocyst

## Abstract

**Background:** Splenic cysts represent a pathology seldom encountered in practice, the most often etiology being, parasitic, congenital and pseudo–cysts, which usually appear post–traumatically or after a splenic infarction.Splenic cysts indicate a surgical treatment when they are large (and thus present a high risk for complications such as rupture, hemorrhaging, compression of the neighboring organs), when they are symptomatic or present complications.

**Case report:** We present the case of a patient diagnosed with splenic cyst in our Clinic, its discovery being incidental, while conducting investigations for an abdominal pain syndrome.

**Discussions:** The clinical case presented completely abides to the literary description–the diagnostic has been incidental, the large size of the cyst has determined the need for surgery (splenectomy), its etiology has been established by means of anatomo–pathological report (the presence of the epithelial inner wall that indicates congenital cysts).

## Introduction

The splenic cysts represent a seldom–encountered pathology, usually their discovery being incidental.  There are two classifications of the splenic cysts: Altemeier and Fowler. According to Altemeier, the splenic cysts are either real cysts (parasitic and non–parasitic) and false cysts or pseudo–cysts (post–traumatic, degenerative, inflammatory) 

According to Fowler, the splenic cysts are both primary cysts (with endocystic epithelial coating) and secondary cysts (without epithelial coating).

**Primary cysts** are also classified as:

1. Congenital serum cysts
2. Post-traumatic cysts (with cellular lining)
3. Inflammatory cysts
4. Neoplazic benign cysts (dermoid, epidermoid, lymphangioma, hemangioma)

**Secondary cysts** are classified as:

1. Traumatic cysts (hematic, serum)
2. Degenerative
3. Inflammatory cysts

This is the most recent classification and it is the one most often used in practice. 

These cysts are diagnosed due to the fact that they trigger splenomegaly or due to the complications that occur firstly from the symptomatic point of view (rupture, hemorrhaging, abscess, etc.)

The treatment is surgical, conducted by splenectomy in the case of symptomatic or complicated cysts. In the event of the incidental discovery, there is a choice between supervision and intervention only in the event of a complication, usually through splenectomy, or conservative interventions (puncture, cystomy, partial splenectomy). 

## Case report–Materials and methods

The patient aged 26, presenting herself to our clinic in January 2010 with non–systematized pain, localized in the epigaster and the left hypochondria. The pain has started a few months before, without being connected to the diet, is of low intensity and of short length (a few minutes). Lately, the painful episodes have increased in number, but the patient has not followed any antialgic treatment. 

With regard to the medical background, what should be taken into account is a vaginal birth 4 years before.

The clinical examination indicates splenomegaly (palpable spleen with 3 cm under the coastal margin), with no other changes aside the ones mentioned. 

The lab exams are within the normal range, including the inflammatory markers (PCR, VSH) and the tumorous ones (CA19–9).

The abdominal ultrasonography indicates the presence of a trans–sonic formation, which occupies the entire left hypochondria, measuring 9/8 cm, entering into contact with the left kidney and the spleen. 

**Figure 1:**
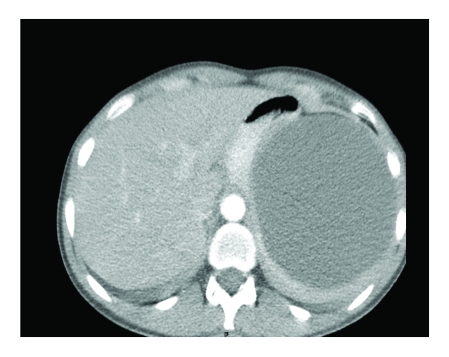
CT Image of the splenic cys

The abdominal CT exam established the diagnostic of splenic cyst (Figure 1)

The CT result: voluminous cystic image with the axial diameter of 11.5/10.7 cm and a cranium–caudal diameter of 8 cm with the departure point at the level of the spleen's superior point and with no actual demarcation limit. The formation pushes the spleen inferiorly and posteriorly and towards the right, the stomach and the pancreas, towards which it presents a demarcation limit. Its content is exclusively liquid and stretches towards the anterior abdominal wall.

The serology for the Echinococcus granulosus has turned negative.

## Results

In conclusion, after conducting all these investigations, the diagnostic for gigantic splenic cyst is set, probably congenital serum primary cyst. 

The patient has been prepared for surgery, due to the existence of the symptomatic gigantic splenic cyst. The surgical intervention has been conducted under general anesthesia with orotracheal intubation, the first incision being a median one, from supraumbilical to infraumbilical.

**Figure 2:**
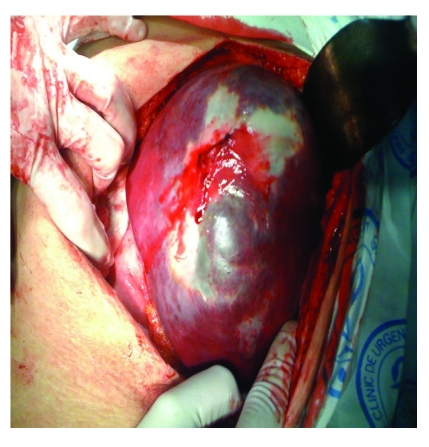
Image taken during surgery after the mobilization of the spleen

**Figure 3:**
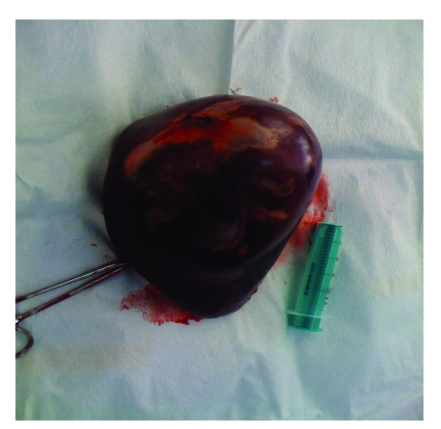
The operated part (the cystic spleen)

During surgery, the presence of the cystic formation localized on the visceral side of the spleen has been noticed (Figure 2), the splenic parenchyma being compressed by the formation and pushed upwards and backwards (Figure 3). The ‘in hil’ splenectomy has been conducted, without the evacuation of the cystic contents. 

Post–surgery, the evolution of the patient has been favorable, being released from hospital 7 days after the surgery, with the recommendation to continue the injectable anti–coagulant treatment afterwards, anti–aggregation, and depending on the thrombocyte number, (the patient has presented post–surgical thrombocytosis up to the value of 1,000,000 plates/mm^3^).

The anatomo–pathological examination has indicated a cystic spleen formation with a wall composed of conjunctive fiber tissue with areas of hyalinization, which contains chronical inflammatory limphoplasmacytic infiltrations. The cystic wall presents epithelium areas of a scuamous type. The histopathological aspect description is compatible with the diagnostic of primary epithelial splenic cyst

The patient has presented herself to the check-up, one month following surgery.

## Discussions

The splenic cysts are a seldom–encountered pathology, usually their discovery being incidental. The most often encountered types are the primary parasitic and congenital cysts, while among the secondary ones; the most frequent are the post–traumatic cysts or the ones developed following an infection or an infraction of the spleen. 

From the international communications up to date, approximately 800 cases of splenic cysts have been diagnosed, among which the majority is parasitic and approximately 300 of them are congenital cysts. The etiological diagnostic of the cysts is not always easy, usually departing from the exclusion of infectious cases (bacterial, parasitic), the exclusion of some traumatisms, neoplastic disorders.  

The majority of the splenic cysts are incidentally discovered, but there are cases in which they might manifest symptoms such as upper abdominal pain, a pain of the left shoulder, and urinary symptoms due to the compression of the left kidney. Some cysts are diagnosed during surgery, either during a surgical intervention for another pathology or on the occasion of a surgical intervention due to a complication of the cyst such as rupture, hemorrhage, abscess. 

The treatment of the splenic cysts remains the subject of controversy in literature concerning the establishment of the surgical indication. There is, however, a consensus, which established the fact that in the case of symptomatic cysts, of the complicated ones, as well as in the case of cysts with a diameter of 4–5 cm, the treatment is surgical. It is possible to conduct splenectomies, partial splenectomies through classical or laparoscopic surgery. 

Supervision is recommended in the situation of the cysts discovered incidentally, with dimensions less than 4–5 cm, the ulterior evolution being impossible to accurately predict. In the event of the development of the symptoms or the increase in dimension, surgical treatment will be applied. Cases describing the extremely rare involution of some small cystic formations have been reported.

## Conclusions

The clinical care previously presented, generally respects the descriptions of the specialized literature. The diagnostic has been incidentally established, during the investigation for painful abdominal syndrome.

The surgical treatment has been the first recommendation, given the size of the cyst and the presence of some symptoms, even attenuated. 

We have favored the open surgical approach given the dimensions of the cyst, the tight connection to the left kidney and the possibility of occurrence of some serious surgical complications, such as hemorrhaging and lesions to the neighboring organs. 

The certified etiological diagnostic has been provided by the anatomo-pathological exam, which has indicated the inner cystic epithelium wrapping. 
